# Detecting Unknown Artificial Urban Surface Materials Based on Spectral Dissimilarity Analysis

**DOI:** 10.3390/s17081826

**Published:** 2017-08-08

**Authors:** Marianne Jilge, Uta Heiden, Martin Habermeyer, André Mende, Carsten Juergens

**Affiliations:** 1 Geomatics/Remote Sensing Group, Geography Department, Ruhr-University Bochum, Universitaetsstrasse 150, D-44780 Bochum, Germany; carsten.juergens@rub.de; 2 German Aerospace Center (DLR), German Remote Sensing Data Center (DFD), Muenchner Strasse 20, D-82234 Wessling, Germany; uta.heiden@dlr.de (U.H.); martin.habermeyer@dlr.de (M.H.); 3 Administrative District Office Zwickau, Department for Surveying, Geodata Management, Scherbergplatz 4, D-08371 Glauchau, Germany; Andre.Mende@landkreis-zwickau.de

**Keywords:** imaging spectroscopy, urban areas, spectral library, dissimilarity, unknown surface materials

## Abstract

High resolution imaging spectroscopy data have been recognised as a valuable data resource for augmenting detailed material inventories that serve as input for various urban applications. Image-specific urban spectral libraries are successfully used in urban imaging spectroscopy studies. However, the regional- and sensor-specific transferability of such libraries is limited due to the wide range of different surface materials. With the developed methodology, incomplete urban spectral libraries can be utilised by assuming that unknown surface material spectra are dissimilar to the known spectra in a basic spectral library (BSL). The similarity measure SID-SCA (Spectral Information Divergence-Spectral Correlation Angle) is applied to detect image-specific unknown urban surfaces while avoiding spectral mixtures. These detected unknown materials are categorised into distinct and identifiable material classes based on their spectral and spatial metrics. Experimental results demonstrate a successful redetection of material classes that had been previously erased in order to simulate an incomplete BSL. Additionally, completely new materials e.g., solar panels were identified in the data. It is further shown that the level of incompleteness of the BSL and the defined dissimilarity threshold are decisive for the detection of unknown material classes and the degree of spectral intra-class variability. A detailed accuracy assessment of the pre-classification results, aiming to separate natural and artificial materials, demonstrates spectral confusions between spectrally similar materials utilizing SID-SCA. However, most spectral confusions occur between natural or artificial materials which are not affecting the overall aim. The dissimilarity analysis overcomes the limitations of working with incomplete urban spectral libraries and enables the generation of image-specific training databases.

## 1. Introduction

Accurate differentiation and identification of urban surface materials is an important requirement for area-wide land cover mapping, and thus for subsequent derivation of further urban data products. Due to their high spectral and spatial information content [1], very-high resolution airborne imaging spectroscopy data have been recognised as a valuable data resource for augmenting surface material inventories [2,3]. Surface material inventories serve as input for various applications, such as urban planning, imperviousness mapping [4,5,6], hydrological modelling [7,8], urban green structure analysis [9,10], and urban climate modelling [11,12,13,14,15,16].

For successful and reliable surface material mapping using very-high resolution airborne imaging spectroscopy data, spectral mixture analysis has been frequently used. Such analysis requires the detection of endmembers that represent the spectrally diverse surface materials and their intra-class variabilities [17,18,19] in a given scene. The correct endmember detection is essential for the subsequent spectral unmixing analysis [20,21]. However, endmember can also be used for classification [22,23,24], or any other data mining methodology [25]. To date, most success has been achieved with image-specific endmembers since they comprise all scene-based structural and compositional information, sensor artefacts, and acquisition-based data characteristics [26].

Manual development of a suitable endmember set is challenging, since urban areas are spectrally very diverse [27,28,29]. Therefore, emphasis has been put on semi-automated empirical approaches, such as the well-known Pixel-Purity-Index method [30]. Fully automated endmember detection algorithms fit a simplex to the point cloud of the data set in the feature space. Examples of these model-based approaches are Minimum Volume Transforms [31] and the N-FINDR. Optimization techniques have been integrated in methods such as Iterative Error Analysis (IEA) or Automated Morphological Endmember Extraction (AMEE) (see [18]). Rogge et al. [32] made use of spatial sub-sampling via local endmember extraction to reduce the size of the original data set. However, the resulting endmembers of these automated algorithms still have to be labelled.

Urban spectral libraries are expert knowledge databases containing the spectral reflectance characteristics of selected artificial surfaces. They have been developed and used across all scales—laboratory [33,34], field [35], and image spectral libraries [36,37,38]. Image spectral libraries used for urban surface material mapping have been demonstrated for Santa Barbara, USA [39], Brussels, Belgium [40], and German cities such as Munich [41] and Dresden [42]. In all cases, more than 20 spectrally different surfaces were detected, comprising biotic and artificial materials. Different colours, coatings, and degradation processes [43] of the materials result in various degrees of spectral intra-class variability. Further variability is introduced by the varying illumination effects [44] resulting from different inclinations of the sensor and the sunlight, and the urban object itself (roof pitch). Intra-class variabilities increase the number of spectrally distinct urban surfaces in very-high resolution airborne imaging spectroscopic data.

The need for image-specific urban spectral libraries is still very high and requires expert knowledge of the characteristics of spectral urban surfaces. In recent years, more attention has been paid to the development and utilization of universal image spectral libraries where a wide range of known urban surface material spectra are generated and stored [25,39]. This progress has evolved owing to the need for area-wide material mappings in diverse geographic regions. That means the inclusion of urban surface materials that characterise regional and cultural trends. Additionally, new urban materials continuously enter the market and their new spectral variations have to be taken into account. The spatial and temporal applicability of existing image spectral libraries is therefore limited.

Based on these observations, it is concluded that it will be impossible to create and maintain a complete and globally applicable spectral library. Imaging spectroscopy techniques are needed that are able to handle the incompleteness of spectral libraries when applied to unknown scenes, and that are also designed to cope with regional-, sensor-, or acquisition-specific characteristics. In this study, a spectral dissimilarity analysis has been developed to aim for a fully automated detection of unknown urban surface materials in high-resolution airborne imaging spectroscopy data using an extensive image library of urban materials. The specific objectives are to:

- Determine unknown scene-based surface material spectra using an incomplete spectral library
- Focus on the detection of pure spectra and avoid detection of spectral mixtures
- Categorise detected unknown surface materials based on spatial and spectral characteristics to support future material-specific identification

Basically, unknown surface materials are identified based on their spectral dissimilarity compared to known library spectra by means of an iterative similarity analysis (Section 2.3.1 and Section 2.3.2). For the spectral dissimilarity analysis, a basic spectral library (BSL) is used that comprises an extensive collection of urban surface materials occurring in Germany. The proposed methodology is suitable for detecting urban surface materials in imaging spectroscopy data that are not yet included in the BSL.

## 2. Methods

**Figure 1:**
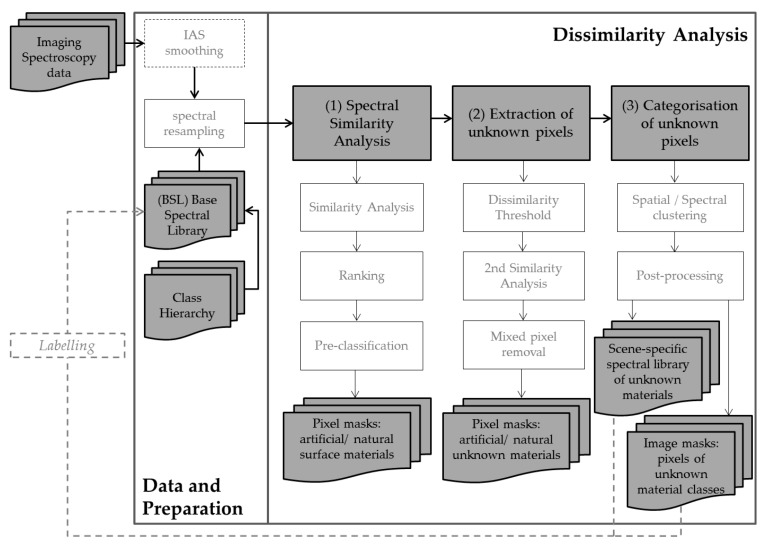
Basic concept of a spectral dissimilarity analysis to detect unknown urban surface materials in high-resolution airborne imaging spectroscopy data.

Initially, three data sets are needed for the dissimilarity analysis, (I) a very-high resolution image (Section 2.1); (II) a basic spectral library (Section 2.2); and (III) a class hierarchy that groups the surface materials in the BSL. The detection of unknown surface materials is based on (1) measuring the similarity between image spectra and library spectra by using a spectral similarity measure (Section 2.3.1); (2) masking pixels with the lowest similarities (high dissimilarities) as potentially unknown surface materials (Section 2.3.2); and (3) categorising the unknown surface materials by a spatial-spectral clustering approach (Section 2.3.3). The procedure also includes two steps to remove mixed pixels from the masks of unknown surface materials. The final result is a scene-specific spectral library with categorised spectrally homogeneous unknown material classes that can serve as a basis for precise labelling of the materials, e.g., by field surveys (Figure 1). The future material-specific identification of detected unknown surface materials is not in the scope of this paper. However, the resulting unknown material classes are spectrally homogeneous and represent predominantly pure image spectra. This means that only one surface material has contributed to the spectral signal of the respective pixel. Thus, the classes can serve as input for further unmixing or data mining techniques, and the detected material classes can be integrated as newly flagged reference spectra into the universal spectral library. Except for the final labelling step, this process is fully automated.

### 2.1. Study Area and Imaging Spectroscopy Data

**Figure 2:**
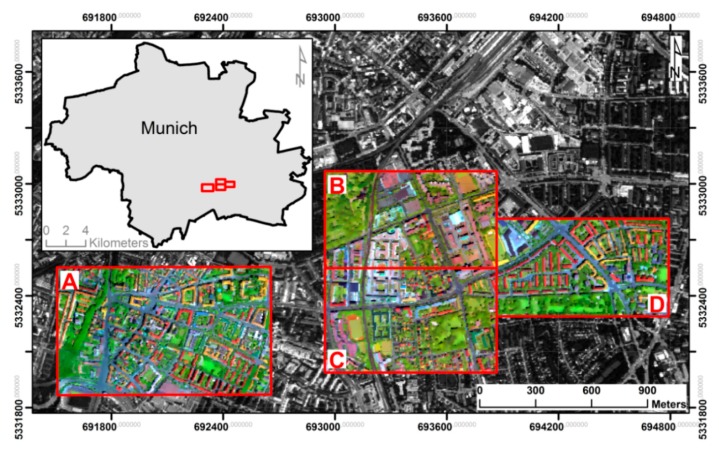
Study area and imaging spectroscopy (HyMap) data for the four test sites A, B, C, and D in Munich, Germany (R = 1652 nm, G = 719 nm, B = 543 nm).

The city of Munich, Germany, was chosen as the study area to demonstrate the functionality of the developed approach. Four municipal areas characterised by diverse urban structures were selected as test sites (Figure 2). The test sites range in function from residential to commercial to industrial to leisure exploitation, and thus include a large variety of surface material classes accompanied by high inter-class variability.

The study area was recorded during the HyEurope2007 campaign on 17 and 25 July using the airborne imaging spectrometer HyMap operated by the German Aerospace Center (DLR) in Oberpfaffenhofen. This sensor records data in 128 contiguous spectral bands between 450 nm and 2500 nm. The flight altitude of about 2000 m resulted in a ground sampling distance (GSD) of 4 m and a swath width ranging from 2 to 2.5 km. A detailed description of the sensor characteristics can be found in [45].

Pre-processing of the data [41] includes correction for radiometric effects according to [45] and the removal of three noisy bands (bands number 1, 33, and 34). The remaining 125 spectral bands were subject to conversion from radiance to surface reflectance values and nadir-normalization by the ATCOR-4 software [46] due to recognition of a brightness gradient. Geometric correction and referencing to the UTM WGS-84 coordinate system was carried out with ORTHO software [47]. Orthorectification was based on the digital terrain model derived from SRTM (Shuttle Radar Topography Mission) data [48]. Mean geometric accuracy was calculated and resulted in 0.8 pixels for the entire data set. For a precise similarity analysis (Section 2.3.1), 12 more spectral bands (from the wavelength ranges 1788 to 2067 nm and 2465 to 2496 nm) were removed due to remaining noise and the presence of atmospheric effects. The final imaging spectroscopic data set contained 113 spectral bands.

### 2.2. Spectral Library Development

For the spectral dissimilarity analysis, an initial spectral library is needed. For setting up the BSL, image spectra were extracted from high resolution imaging spectroscopy data (HyMap) acquired over the German cities of Dresden, Potsdam [37], and Munich (Table 1).

Based on these diverse data sets, spectral variations resulting from different illumination and observation conditions [44], regional characteristics, and data processing are considered. Accordingly, the BSL contains all the surface materials occurring in the test sites to the best of the authors’ knowledge, with special emphasis on artificial materials. However, due to phenological variations, different vegetation types and soils are underrepresented in the BSL.

Image spectra per material class were selected based on the hierarchical categorisation scheme introduced in [37]. Therefore, spectra for each surface material class were determined by defining regions of interest in the images (Table 1) based on field investigations, spectral expert knowledge, infrared aerial imagery, and Google image products. In order to use image spectra as reference spectra, spectral purity was ensured by selecting homogeneous areas while excluding boundaries and small urban objects. Finally, the library was manually inspected and reduced by eliminating any potentially remaining mixed material spectra, resulting in almost 5200 BSL spectra organised in 23 surface material classes that are further divided in 8 natural, 14 artificial, and one class of shadow. The shadow class is determined by image pixels collected over shaded natural and artificial surfaces. This class is used for excluding shaded regions in the dissimilarity analysis. An overview of included reference spectra per surface material class is given in Table 2.

### 2.3. Dissimilarity Analysis

The dissimilarity analysis comprises three main processing steps that are outlined in Section 2. Further, as illustrated in the concept (Figure 1), two pre-processing steps, IAS smoothing and spectral resampling, are included. The first pre-processing step is optional and accounts for the high spectral intra-class variability of urban surfaces that can be referred to spectral variations, as described in Section 1, and image noise. The iterative adaptive smoothing (IAS) filter [49] reduces the image noise while retaining the object edges. The second pre-processing step incorporates a spectral resampling of the BSL to the spectral resolution of the imaging spectroscopy data used, in order to make processing independent of the relevant characteristic in the image data. The spectral resampling of the BSL is based on interpolating the spectra to the wavelength information of the input imaging spectroscopy data.

#### 2.3.1. Spectral Similarity Analysis (1)

The spectral dissimilarity analysis starts with a quantitative analysis of spectral similarities among all image spectra (Section 2.1) and all available BSL spectra. Over the past decades a wide range of similarity measures have been developed to numerically evaluate the match between two spectra. These include, for instance, the well-known Spectral Angle Measure (SAM) [50], Spectral Information Divergence (SID) [51], Spectral Correlation Angle (SCA) [52], and Spectral Correlation Measure (SCM) [53]. Most of these measures evaluate the match based on the spectral shape while ignoring the amplitude of the spectra. Additionally, hybrid similarity measures have been developed to exploit the advantages or minimise the weaknesses, of two similarity measures. The Jeffries–Matusita-Spectral Angle (JM-SAM) is one such hybrid approach that was developed for mangrove applications [54]. Naresh Kumar et al. [55] developed and compared SID-SCA with SID-SAM [56] and found higher performance of the SID-SCA in the visible wavelength range targeted to a discrimination of *vigna* species using laboratory measurements. The efficiency of specific similarity measures was analysed in [55,57,58], with SID-SCA being considered the best performing. Accordingly, SID-SCA was selected as the most appropriate technique for the spectral similarity analysis in this study. However, to the authors’ knowledge, none of the similarity measures are specifically adapted to urban surface discrimination.

For the quantitative analysis of spectral similarities using SID-SCA, each pixel spectrum is compared with each BSL spectrum, resulting in *n* similarity values, where *n* is the total number of BSL spectra. Similarity values are normalised and inverted to enable a logical interpretation, meaning that similar spectra have similarity values close to 1 and dissimilar spectra have similarity values close to 0.

In the next step, the *n* similarity values per pixel are ranked in descending order (similar to dissimilar). The best match is represented by the highest obtainable similarity value. However, in this study the ten highest ranked similarity values are used to define the statistically dominant class according to [59]. In this case, the material classes occurring in the ranking of the first ten similarity values are linearly weighted on the basis of the total number of representatives per material class (Table 2), in order to equally consider over- and under-representation of single material classes. Weights of material classes are multiplied by the number of materials classes represented in the ranking and summed up to determine the overall weight of an observed pixel. Finally, the statistically dominant class is represented by the highest percentage obtained for the material weight divided by the summed overall weights.

In summary, for each pixel a single similarity value was defined that enables its assignment to one respective surface material class (Table 2). That allows for a simple separation of the pixels into the coarse material groups artificial and natural surfaces (artificial and natural masks) and shadow. This ranking and pre-classification is crucial for the subsequent extraction and later categorisation of unknown, predominantly pure pixels (Section 2.3.2 and Section 2.3.3).

#### 2.3.2. Extraction of Unknown Pixels (2)

Extraction of unknown pixels is based on the idea that pixels with very low similarity values are not represented in the BSL. Such pixels commonly comprise spectral mixtures as well as predominantly pure unknown materials. A dissimilarity threshold was introduced to distinguish known from unknown pixels and expressed as a percentage of the total number of image pixels. The threshold is applied separately to the artificial and to the natural materials mask, while pixels pre-classified as shadow are neglected. The resulting potentially unknown pixels are stored in two masks, one for pixels with unknown artificial materials and one for those with unknown natural materials. The separation of artificial and natural pixels is important owing to the underrepresentation of natural material classes in the BSL, as described in Section 2.2. Without the separation, natural surfaces are more commonly detected as potentially unknown pixels. This study solely focused on the pixel mask for potentially unknown artificial materials.

In the first instance, a fixed dissimilarity threshold is used to separate unknown from known pixels. However, when using a fixed dissimilarity threshold, a hard boundary for separating similar from dissimilar pixel spectra is set. This results in ignoring dissimilar pixel spectra with low similarity values, but not low enough to be considered as potentially unknown. These outliers are considered in a second similarity analysis, where all image spectra are compared to each of the potentially unknown pixel spectra extracted with the dissimilarity threshold. This comparison follows the same procedure as described in Section 2.3.1. Pixels that are more similar to the potentially unknown pixel spectra than determined by the first similarity analysis are subsequently added to the mask of potentially unknown pixels. The influence of the dissimilarity threshold and the subsequent second similarity analysis are analysed in Section 3.3 and Section 3.4.

Potentially unknown pixels may also comprise spectral mixtures that need to be removed. Single pixels and the border pixels of pixel clusters commonly consist of spectral mixtures. To remove them a 3 × 3 pixel moving window is applied following von-Neumann criteria [60] for analysing neighbourhood relationships. Pixels are considered to be a mixture if an observed mask pixel is not surrounded by at least four direct mask pixels. The remaining pixels represent the mask of unknown and predominantly pure artificial pixels.

#### 2.3.3. Categorisation of Unknown Pixels (3)

In the third step of the dissimilarity analysis, the mask of unknown and predominantly pure artificial pixels is used to build spectrally homogeneous clusters to facilitate a future material based labelling of the unknown pixel spectra. Initially, spatial clusters are built using the von-Neumann criteria. Clusters are then spectrally re-organised based on their internal and external spectral homogeneity. For spectral homogeneity assessment, SAM [50] was used due to the easy interpretation of the results and the high level of experience with this approach in the scientific community. For analysing the internal spectral homogeneity of a spatial cluster, all pixels within this cluster are compared to each other. If inverted similarity values (see Section 2.3.1) exceed an internal homogeneity threshold of 0.9 (radian measure), which is specified according to an inverted threshold of 0.1 [59], a new subcluster is built. An external spectral homogeneity analysis between the newly generated spatial clusters first determines the mean reflectance spectra of each cluster and second, makes a spectral comparison between clusters. Accordingly, clusters are aggregated if the determined similarity values do not exceed a radian measure of 0.9 [59] analogous to the internal homogeneity threshold. Resulting spatially and spectrally homogeneous clusters are subsequently assumed to represent individual unknown surface material classes. However, empirical tests reveal that an additional step (post-processing) is required to ensure the spectral purity of the derived unknown surface material classes. For this purpose, spatially isolated pixels of a single unknown material class are removed according to the single pixel removal method described in Section 2.3.2. Additionally, clusters with fewer than four pixels are deleted, since it is very likely that they still contain spectral mixtures or do not sufficiently represent a new material class.

The remaining pixels represent the final unknown surface material classes detected in the image. The categorisation step results in a scene-specific spectral library of unknown material classes, tagged with respective geographic coordinates, and an image mask of unknown material classes (Figure 1). Extracted unknown material spectra can be labelled and subsequently included in the BSL.

### 2.4. Experimental Setup

The functionality and effectiveness of the described dissimilarity analysis (Section 2.3) were tested using HyMap data for Munich, Germany (Section 2.1). Basically, two setups were designed, a library setup and a dissimilarity threshold setup. For the library setup, specific surface material classes and their respective instances are removed from the universal spectral library to simulate its incompleteness. Subsequently, whether the removed material classes could be detected as unknown surface material classes is tested using the dissimilarity analysis described in Section 2.3. For this purpose, four different BSL cases have been defined:

(1) the BSL is fully applied and assumed to be complete for the respective test sites (*full*)
(2) all instances of the material class roofing tiles are removed (*without tiles*)
(3) all instances of the material class roofing zinc are removed (*without zinc*)
(4) all instances of the material classes roofing tiles and zinc are removed (*without zinc and tiles*)

In library setup (2) roofing tiles are removed since they are a frequently occurring roofing material in German cities and are also observable in other countries. The roofing tile class has numerous spectral signatures because of the huge variety of material characteristics (colour, coating, etc.). In setup (3) zinc is removed, which is relatively unique due to its characteristically wide and deep absorption feature at 1020 nm, which makes it easily distinguishable. Additionally, setup (4) tested how the methodology handles the removal of more than one material class by removing both roofing tiles and zinc.

For the dissimilarity threshold setup, different percentage values, 1%, 2%, 3%, and 5%, are used to determine the mask of potentially unknown surface materials (Section 2.3.2). The impact of the dissimilarity threshold should be analysed regarding (1) the number of detectable unknown surface material classes; (2) the amount of spectral mixtures handled in the analysis, and (3) the influence on the final unknown surface material classes. The different library and dissimilarity threshold setups are individually applied to the four test sites (Section 2.1), producing a variety of outcomes.

### 2.5. Validation

Validation was carried out of the pre-classification of artificial and natural masks and of the detected unknown surface materials. For validating the pre-classified images, the determined statistically dominant material classes and the validation data were pooled into two groups, natural surfaces and artificial surfaces, on the basis of the utilised class hierarchy (Section 2.2). Validation comprises kappa statistics [61], overall accuracies, and producer- and user-accuracies for summarised natural and artificial material classes resulting from test site specific confusion matrices.

The validation data is also used for evaluating detected unknown surface material classes (Section 2.3.3). The spatially and spectrally aggregated unknown material clusters are compared for their spatial agreement with the validation data. For this purpose, unknown surface material classes are labelled manually based on expert knowledge and previous studies [41]. Accuracy is determined by calculating the percentage share of detected clusters and validation clusters.

**Figure 3:**
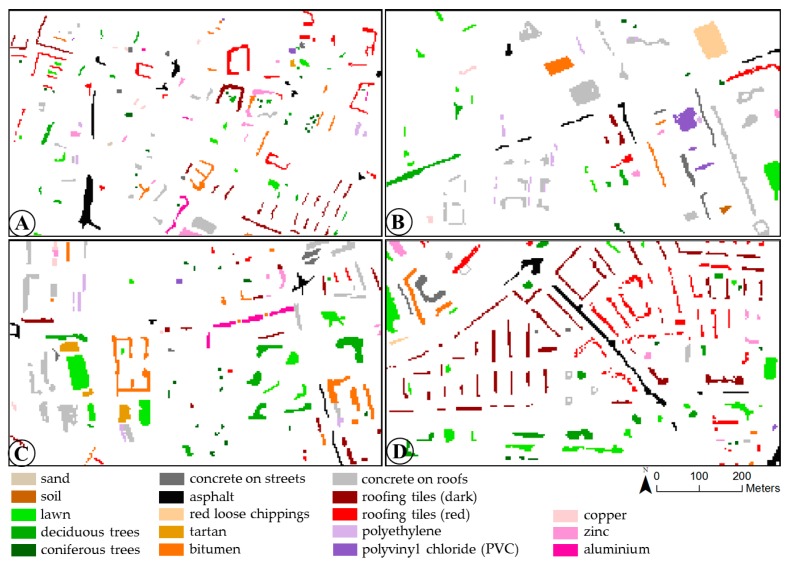
Validation data for test sites (**A**–**D**) in Munich, Germany.

Validation data rest upon digitised building blocks that have been used and described in [41]. The building blocks were manually digitised by means of orthophotos. Surface material classes (Section 2.2) were identified and manually assigned with spectral expert knowledge and field surveys. The underlying orthophotos were simultaneously acquired with a 3K-camera during the hyperspectral flight campaign and had a spatial resolution of 50 cm [62]. At least one digitised building block is present in each of the four test sites. Validation data for the purpose of this study are enhanced by manual selection of single object pixels on the basis of spectral expert knowledge. When selecting pixels as validation data, the spectral intra-class variability of the material classes occurring in the test sites was taken into account as accurately as possible. Regarding the experimental results (see Section 3 and Section 4), validation data for roofing tiles are divided into two colour categories (dark roofing tiles and red roofing tiles). Figure 3 shows the validation data for each of the four test sites.

## 3. Results and Preliminary Assessment

The methodological steps described in Section 2.3 are individually applied to the four test sites (Section 2.1) according to the experimental setup (Section 2.4). The results of the three main steps, comprising pre-classification, masking of unknown predominantly pure pixels, categorising unknown material classes, and evaluating unknown material classes, is shown separately in Section 3.1, Section 3.2, Section 3.3 and Section 3.4.

### 3.1. Pre-Classification (Step 1)

Pre-classification categorises image pixels into natural and artificial surfaces based on the statistically dominant surface material class (Section 2.3.1). The accuracy assessment was primarily done per surface material class (Table 2) to investigate potential confusion between single classes. Further, it has to be mentioned that validation of pre-classification results focusses on assessing the accuracies of predominantly pure surface materials, because the area-wide interpretation of spectral mixtures is not within the scope of this paper. The results of the accuracy assessment are listed in Table 3.

In general, kappa statistics show values between 0.80 and 0.93, with the best result for test site C. Overall accuracies range from 83.14 to 94.24%. The lowest user and producer accuracies were assessed for the artificial pixel mask of test site D. Accuracies are extensively analysed by inspecting confusion at the material level based on the respective confusion matrices. It reveals that confusion mainly occurs between materials within one of the two broad classes—natural and artificial surfaces. Confusion of spectrally similar material classes, such as asphalt and concrete (test site C), or between roofing tiles and red loose chippings, is well-known and documented by other studies [37]. Confusion among artificial and natural material classes is rare. An exception is bright sand that has been also identified as concrete. This is because sand (quartz) is one of the main components of concrete [37].

Results of the accuracy assessment demonstrate the suitability of the presented approach for distinguishing between the two broad classes (a) natural and (b) artificial surfaces. The following analysis focusses on artificial surfaces.

### 3.2. Mask of Unknown Artificial Pixels (Step 2)

**Figure 4:**
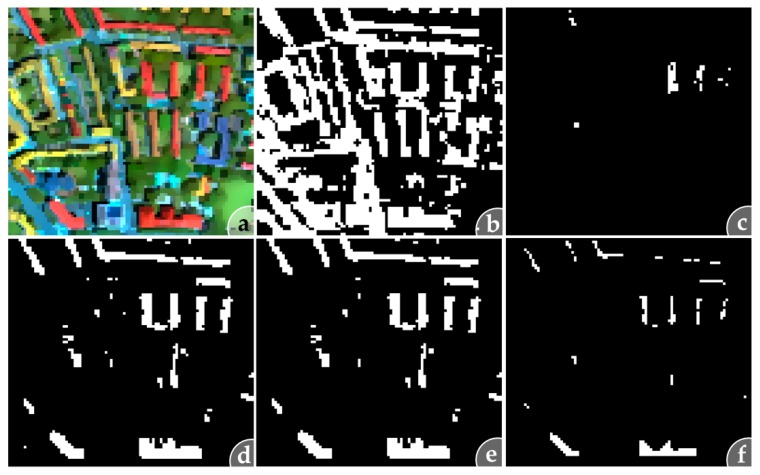
Masking steps to determine unknown artificial pixels for (**a**) a subset of test site D using the BSL without tiles by (**b**) separating artificial pixels from pre-classification results; (**c**) extracting dissimilar artificial pixels based on a 1% dissimilarity threshold; (**d**) enhancing the mask by a second similarity analysis to include outliers; (**e**) removing single pixels and (**f**) removing border pixels based on the von-Neumann criteria for eliminating mixed pixels.

In Figure 4 each single masking step to extract unknown predominantly pure artificial pixels, described in Section 2.3.1 and Section 2.3.2, is graphically illustrated for a subset of test site D.

The results for all test sites are described in terms of the varying library setups and increased dissimilarity thresholds. First, the number of pre-classified artificial pixels is influenced by the library setup applied that imitates the level of incompleteness of the BSL. Artificial masks (Figure 4b) determined from libraries without tiles are generally smaller than masks resulting from libraries without zinc. This is reasonable, since the roofing tile class contains many more instances (589) than the zinc class (143) (see Table 2). Which library setup is used also influences the number and representation of the detected unknown surface material classes. The higher the incompleteness of the library with respect to a given test site, the more pixels are classified as unknown there (Figure 4f). This finding is demonstrated in Figure 4, which illustrates the application of the algorithm on the test site D subset. This test site is characterised by a large number of buildings covered with roofing tiles and shows a high number of detected unknown pixels for the library set “without tiles”. However, it also shows that some roofing tile pixels expected to be unknown are missing by comparing the artificial mask (Figure 4b) and validation data (Figure 3) with the resulting unknown pixel mask (Figure 4f).

**Figure 5:**
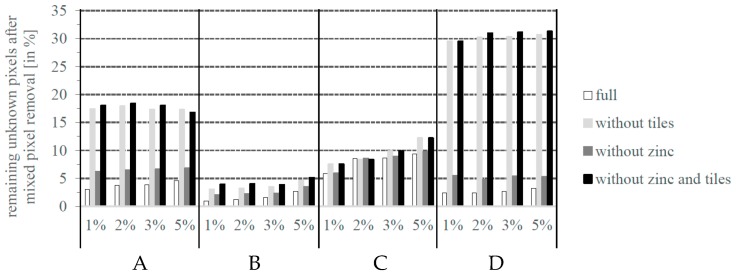
Remaining unknown artificial pixels after single and border pixel removal.

Second, an increasing dissimilarity threshold causes an increase in detected potentially unknown pixels after the second similarity analysis. In general, the mask of potentially unknown pixels contains new unknown classes, more variability (instances) of these classes, and spectral mixtures. Especially the number of spectral mixtures needs to be monitored in more detail (Section 3.3). In all test sites and experimental settings mixed pixel removal (Section 2.3.2) results in a rather massive decrease of potentially unknown pixels and manifests the impact and importance of this step. It can be assumed that most of the spectral mixtures are excluded from the mask of unknown pixels except from those spectral mixtures that are unique (see Section 3.3). Further, a general slight increase of pixels detected as unknown can be observed with a rising dissimilarity threshold (Figure 5). Given a successful mixed pixel removal, it can be assumed that increasing dissimilarity thresholds integrate more spectral variability or instances of unknown materials.

### 3.3. Categorisation (Step 3)

**Figure 6:**
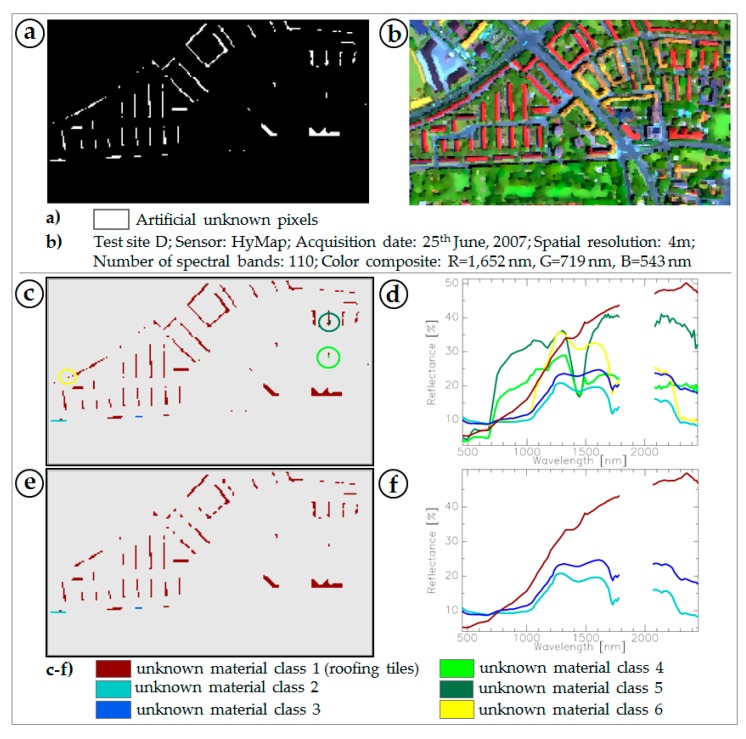
Extraction of unknown artificial material classes from the (**a**) mask of unknown artificial pixels determined from the (**b**) image data of test site D with a BSL setting without roofing tiles and a dissimilarity threshold of 1%. Unknown artificial pixels are subject to (**c**) spatial and spectral clustering to identify spectral mixtures from (**d**) mean unknown class reflectance, accompanied by (**e**) post-processing to delete unknown material classes of remaining mixed pixels to result in an (**f**) scene-specific spectral library of unknown artificial material classes.

Detected unknown pixels (Section 2.3.3 and Section 3.2) are finally categorised based on spatial and spectral metrics to support subsequent labelling and integration into the BSL. This step also includes further revision regarding potentially remaining mixed pixels. In Figure 6 the functioning principle is shown in test site D for a spectral library setting without tiles and an applied dissimilarity threshold of 1%.

The mask of unknown artificial pixels (Figure 6a) shows homogeneous spatial clusters that correspond well with urban objects in the image data (Figure 6b). According to Section 2.3.3 clusters are re-organised in terms of spatial and spectral homogeneity (Figure 6c). Categorised clusters (unknown material classes) show a detailed separation and aggregation of distinct homogeneous urban objects (Figure 6c). The respective mean reflectance spectra for each unknown material class are given in Figure 6d. Visual comparison of the mean reflectance spectra shows high similarity with mean spectra of roofing tiles in the validation data (Figure 3). However, visual comparisons of Figure 6a,c with the roofing tile classes of the validation data (Figure 3d) point out absent unknown mask pixels in the region of objects covered by red roofing tiles (Section 3.2). This accords with the known issue of spectral similarities between red roofing tiles and red loose chippings [37]. Accordingly, pixels representing red roofing tiles were not considered in the mask of potentially unknown pixels. In addition, five more unknown mean reflectance spectra (Figure 6d) do not correspond with the validation spectra. Visual inspection in combination with spectral expert knowledge revealed that amongst the identified roofing tile class spectral mixtures also remain (light green, dark green, yellow in Figure 6d). Their spatial appearance (Figure 6c) demonstrates that most of these spectra occur as single pixels without being attached to homogeneous clusters. Consequently, to ensure that only predominantly spectrally pure pixels are in the unknown material class, post-processing to remove the remaining mixed pixels from the mask of categorised pixels clusters was carried out. This results in three unknown material classes: the already identified unknown material class (roofing tiles), and two further unknown material classes (light and dark blue) that are displayed in Figure 6e. Corresponding mean reflectance spectra are given in Figure 6f. Despite the fact that the two remaining unknown material classes are characterised by a similar spectral shape, the main variations dominate the NIR and SWIR region in terms of amplitude differences, which is the decisive factor for separating the two classes. A visual inspection of the two unknown material classes with a very high-resolution image, e.g., image products from Google Earth, reveals that both classes feature solar panels. Although the BSL is an extensive collection of reference spectra, it so far lacks solar panels. First angular-dependent spectroscopic measurements (goniometer) have already shown that the spectral signature of solar panels is highly influenced by the observed azimuth. Consequently, a separation into two material classes is reasonable due either to a different construction type or to a varying inclination angle while acquiring spectral information. In addition to the detection of previously removed material classes (Section 2.4), two new unknown material classes identified as solar panels were found and confirm the efficiency of the method.

**Figure 7:**
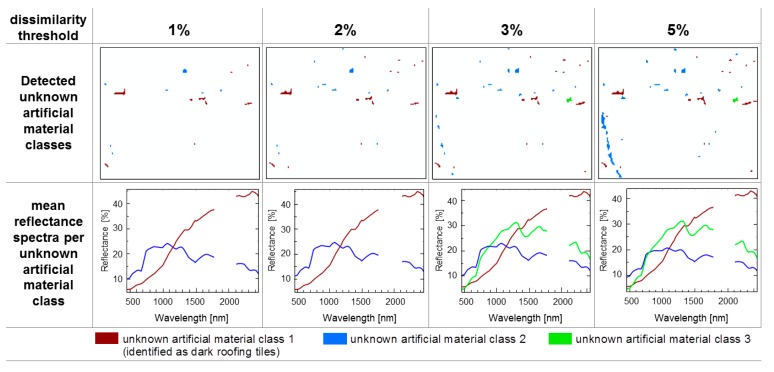
Impact of an increasing dissimilarity threshold on the number of detected unknown artificial material classes elucidated for test site C with a BSL without tiles.

The sensitivity of the dissimilarity threshold regarding the number of detected and categorised unknown material classes is shown in Figure 7 for test site C. As described in Section 3.2, the increase in the dissimilarity threshold results in an increase in the mask of unknown pixels. Moreover, the number of spectrally homogeneous material classes and the number of pixels representing the classes both increase.

Visual inspection of the mean reflectance spectra and Google Earth images reveals that an unknown material class 1 (brown) could be identified as dark roofing tiles. Unknown material classes 2 (blue) and 3 (green) could be assigned as a greened roof (green) and parts of a partially greened tramline (blue). Both unknown material classes (green and blue) are not pure from a spectral point of view. The spectral signature of the greened roof is composed of photosynthetically active vegetation and the underlying substrate, whereas the tramline spectra results from a mixture of gravel, steel rails, and some vegetation fractions. Although the methods introduced in Section 2.3.2 and Section 2.3.3 focus on removing mixed pixels, unknown mask pixels composed of spectral mixtures remain as long as they are spectrally unique and appear as large homogenous objects, such as large roofs or the track of a tramline. Also noticeable is the consistency of the spectral representation of the detected unknown material classes, which seems to be independent of the number of unknown material class pixels involved in the mean reflectance calculation.

### 3.4. Validation of Detected Roofing Tiles and Zinc Material Classes

**Figure 8:**
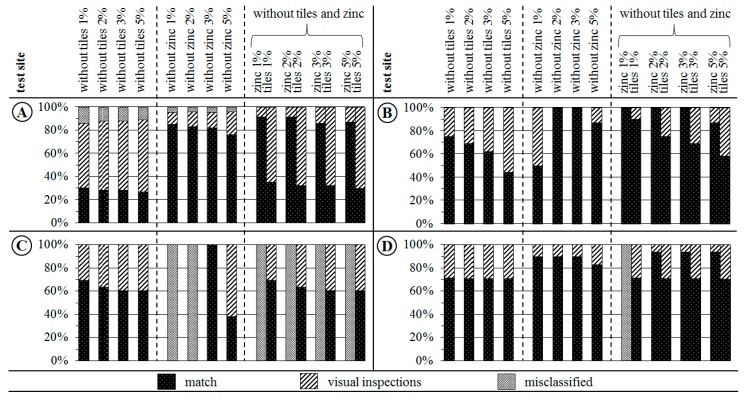
Percentage of spatial agreement by validating all pixels of an unknown material class (columns) detected for different library setups in the four test sites (**A**–**D**). Validation results are composed of a spatial match (black) of unknown pixels with validation pixels of material classes zinc or dark roofing tiles, visual inspections (hatched) by visual comparisons of mean spectra due to missing validation data, and (grey) misclassifications or missing data for an unknown material class.

The spectral and spatial representation of detected unknown materials that have been removed from the BSL (dark roofing tiles, zinc) are validated based on visual inspections and using reference data described in Section 2.5. Results of the quantitative accuracy assessment are summarised in Figure 8. The validation (Figure 8) is mainly based on a simple spatial match of pixels belonging to an unknown material class with validation pixels of previously erased material classes. The mean spectra of the remaining unknown material class pixels were individually validated by visual comparisons (visual inspections) with the mean spectra of the respective validation class and by spectral expert knowledge. Accuracies determined by the two validation methods (match and visual inspection) are equally assessed. Consequently, the overall accuracy of detected unknown material class pixels is indicated by a combination of accuracies obtained from spatial match and visual inspection (Figure 8).

Generally, unknown material classes that were removed from the BSL could be re-detected as spatially and spectrally homogeneous pixel clusters. The agreement mainly varies with the test site and library setting. The number of misclassified unknown material classes is small except for test site C. Usually the match (black column) of the results with validation data slightly decreases with an increasing dissimilarity threshold. Hence, for determining the overall accuracy, the percentages of match and visual inspection need to be considered.

A more precise inspection of Figure 8 demonstrates good (test site A) to very good matches (test sites B and D) for different library setups. However, in test site C zinc could not be detected by applying a dissimilarity threshold of 1% or 2%. Inspection of the results reveals that zinc image spectra were spectrally not dissimilar enough to be added to the mask of potentially unknown pixels. In the pre-classification, urban objects covered by zinc are assigned to the material class aluminium. However, by increasing the dissimilarity threshold (3%, 5%) zinc was correctly detected.

Further, the results indicate that the more material classes missing in the spectral library, the more challenging their detection. Despite an increased level of library incompleteness, in general, the missing surface materials could be detected with the exception of test site C. Simultaneously, an increased level of library incompleteness results in less spectral variation per unknown material class because of the fixed percentage of image pixels that are flagged as unknown (dissimilarity threshold). Additionally, the detection of unknown material classes is also influenced by the number of pixels per material class in the test site image and the level of dissimilarity among distinct unknown classes. Underrepresented unknown material classes are not considered as potentially unknown if spectra of another unknown class are more dissimilar and the percentage amount (dissimilarity threshold) of dissimilar spectra is reached.

## 4. Discussion

Functioning, benefits, and drawbacks of spectral dissimilarity analysis (Section 2.3) are discussed in the following. The above results have confirmed the functioning of re-detection of previously removed material classes (Section 3.3 and Section 3.4). Completely new surface materials (solar panels and tram rail tracks) could be detected and identified as discrete classes (Section 3.3). The developed methodology was extensively tested on four test sites with different settings for the BSL and varying dissimilarity thresholds (Section 2.4).

The findings reported in Section 3.1 and Section 3.2 demonstrate the importance of the applied similarity measure SID-SCA. This measure is essential for the steps of pre-classifying the image, determining dissimilar pixel spectra for building a mask of potentially unknown pixels, and enhancing the potentially unknown pixel mask by means of a second similarity analysis. Pre-classification results are very promising as to the separation of natural and artificial pixels (Table 3). However, a detailed material-based accuracy assessment (Section 3.1) reveals the limits of the SID-SCA approach. Spectral confusion of different materials was observed, such as between zinc and aluminium. Both materials are characterised by distinct broad absorption features, but their absorption maxima differ only slightly, by about 140 nm (Section 3.4). Based on SID-SCA, zinc pixels (test site C) are too similar to the aluminium class and thus are not added to the mask of potentially unknown pixels. Another example reveals the importance of considering amplitude as a spectral feature. Besides spectral absorption features, amplitude is the dominant spectral feature for differentiating asphalt and concrete. Previous studies [37,63] have already reported the importance of spectral features, the shape of a spectrum, and the amplitude for identifying urban surfaces. In [58] a hybrid similarity measure that fuses shape and amplitude features (Fusing SAF) was developed. The application of this measure (Fusing SAF) could provide enhanced results for material mapping of spectrally similar shaped material classes in urban areas. However, both cases of spectral confusion do not affect the aim of the important pre-classification step itself, since the confusion appears within the same material group (artificial or natural).

The pre-classification results are the basis for the subsequent extraction of potentially unknown predominantly pure pixels. The number of extracted potentially unknown pixels using a fixed dissimilarity threshold is increased by converting the dissimilarity threshold to a scene-based threshold and applying a second similarity analysis (Section 2.3.2 and Section 3.3, Figure 4). This way, the fixed dissimilarity threshold is adapted to the characteristics of the image data used. In general, it was found that the number of potentially unknown pixels increases with an increasing dissimilarity threshold (Section 2.4). Findings from Section 3.2 indicate that unknown material spectra are already detectable with a dissimilarity threshold of 1%. A further increase in the dissimilarity threshold is typically associated with the formation of larger spatial clusters and usually results in more spectral variations of unknown material classes. Consequently, a larger dissimilarity threshold can be useful for a more incomplete spectral library. However, an increasing dissimilarity threshold is frequently associated with an increase in the number of finally remaining spectral mixtures (Section 3.2), which needs to be considered. On the other hand, spectral mixtures that build large spatial clusters such as vegetated roofs or tram rail tracks remain, despite the mixed pixel removal steps which are generally based on spatial constrains (single pixels, object border pixel).

The number of mixed pixels also correlates with the size of the urban objects and the associated GSD of the image. In this study, airborne imaging spectroscopy data with a GSD of about 4 m were used. By analysing different test sites characterised by different object sizes, we could observe how the performance of the approach declines for test site C, which contains the smallest urban objects and thus fewer pure, surface material spectra. However, except for test site C, mixed pixel removal was successful. An additional test concerning the spectral purity of detected unknown surfaces, e.g., using iterative endmember selection [64] would be an important task for future studies. With the presented methodology, it will be up to the user to decide whether a predominantly pure material has been identified, or not.

## 5. Conclusions and Outlook

Image-specific urban spectral libraries are widely and successfully used in urban imaging spectroscopy studies. In this paper, a methodology is presented that can handle the incompleteness of spectral libraries. The proposed spectral dissimilarity analysis was developed to (1) detect image-specific unknown urban surface materials while (2) avoiding spectral mixtures, and to (3) categorise detected unknown surface materials, e.g., to support a material specific identification. The fundamental approach is based on the assumption that unknown surface materials are dissimilar to known spectra provided in the BSL. Dissimilar image spectra are extracted by means of a scene-based threshold applied on previously determined spectral similarity values resulting from SID-SCA analysis. Potentially unknown image spectra are separated from mixed pixels by spatial and spectral metrics. In a final step, the remaining dissimilar image spectra are categorised to build spectrally homogeneous material clusters.

The efficiency of the approach is demonstrated by applying it to different test sites, distinct dissimilarity thresholds, and to different cases of an incomplete spectral library. The incompleteness is simulated by removing material classes from an initial spectral library (BSL) with the aim to detect these classes again as unknown scene-based surface materials. In nearly all cases, the results indicate the successful re-detection of unknown surface materials using spectral dissimilarity analysis. Limitations are associated with the degree of incompleteness. It is shown that with a higher incompleteness of the BSL, unknown material classes are more reliably detected with a higher dissimilarity threshold. Beside the increase of spectral variabilities of detected unknown material classes, an increasing dissimilarity threshold is also associated with a gain in the number of remaining mixed pixels. It can be concluded that the dissimilarity threshold needs to be precisely adjusted based on the level of incompleteness of the BSL in order to detect unknown materials or material instances, and to keep the number of remaining spectral mixtures down. Finally, it will be up to the user to find a trade-off between high spectral variability and a low number of remaining mixed pixels. However, even a small set of representative unknown predominantly pure pixels can be useful for applying any subsequent image analysis techniques. As an example, in [42] rare spectral representatives, seedlings, are used to further enhance the endmember set for area wide surface material identification in urban areas.

The suitability of the presented methodology is further underlined by the detection and identification of unique material classes such as solar panels, which were identified as a completely new surface material with respect to the BSL. Comparing the material classes represented in the initial BSL with other urban spectral libraries [34,35], it can be assumed that the application of this BSL to cities in the USA and Great Britain could be promising. However, it needs to be tested for other geographical regions, where more unknown surface materials can be expected. Another important aspect that should be addressed in future research on the applicability of the method is the GSD of the imaging spectroscopy data needed to obtain pure representatives of all surface materials of interest.

The high number of different surface materials and respective variations in urban areas hampers the use of spectral libraries and the transferability of library-based technologies for untested geographic regions. The developed methodology is a first step toward overcoming this limitation. It can be used to create image-specific training databases and it can also serve as a technology for enlarging urban spectral libraries to make possible their widespread utilisation. In the future, the technique will be enhanced in order to test the spectral purity of the unknown material classes and also to identify all spectrally pure surface materials in a high resolution imaging spectroscopy data set of an urban area.

## Figures and Tables

**Table 1:** Airborne imaging spectroscopy data used for the extraction of reference spectra for the BSL.

Acquisition Date (DD-MM-YYYY)	Test Site	Pixel Size	No. of Bands
18-05-1999	Dresden	7.7 m	128
18-05-1999	Potsdam	4.0 m	128
01-08-2000	Dresden	3.3 m	126
20-07-2003	Dresden	3.5 m	126
17-06-2007	Munich	4.0 m	125
25-06-2007	Munich	4.0 m	125

**Table 2:** Class hierarchy to separate BSL spectra of material classes (their occurrence is in brackets) into artificial and natural surface material groups.

Artificial		Natural
Paving and Open Space Materials	Roofing Materials	
asphalt (339) synthetic turf (264) tartan (75) paving concrete (167) red loose chippings (161)	roofing aluminium (181)	sand (31)
	roofing bitumen (400)	soil (96)
	roofing concrete (352)	coniferous tree (248)
	roofing copper (164)	deciduous tree (277)
	roofing polyethylene (358)	lawn (434)
	roofing polyvinyl chloride (PVC) (231)	pond (183)
	roofing tar (15)	pool (34)
	roofing tiles (589)	river (354)
	roofing zinc (143)	

Additional 97 reference spectra of shadow have been integrated in order to avoid shaded areas in the artificial and natural pixel mask (Section 2.3.1 and Section 2.3.2).

**Table 3:** Pre-classification accuracies for the test sites (column 1) using the full BSL setting comprise grouped producer and user accuracies within natural (columns 2–3) and within artificial material classes (columns 4–5) demonstrating the general separation of the broad classes natural and artificial. Overall accuracies (column 6) and kappa statistics (column 7) reveal the general pre-classification accuracies of single material classes.

Test Site	Producer Acc.-Natural	User Acc.-Natural	Producer Acc.-Artificial	User Acc.-Artificial	Overall Accuracy	Kappa Statistic
A	89.89%	83.98%	91.60%	90.92%	92.32%	0.91
B	93.15%	93.30%	86.95%	89.25%	83.14%	0.80
C	95.81%	90.63%	92.91%	93.13%	94.24%	0.93
D	93.07%	94.54%	78.83%	74.17%	86.22%	0.81
